# Medical and Social Determinants of Subsequent Labour Market Marginalization in Young Hospitalized Suicide Attempters

**DOI:** 10.1371/journal.pone.0146130

**Published:** 2016-01-19

**Authors:** Thomas Niederkrotenthaler, Petter Tinghög, Sidra Goldman-Mellor, Holly C. Wilcox, Madelyn Gould, Ellenor Mittendorfer-Rutz

**Affiliations:** 1 Medical University Vienna, Center for Public Health, Institute of Social Medicine, Suicide Research Unit, Vienna, A-1090 Vienna, Austria; 2 Karolinska Institutet, Department of Clinical Neuroscience, Division of Insurance Medicine, 17 177 Stockholm, Sweden; 3 The Swedish Red Cross University College, Stockholm, Sweden; 4 University of California, Merced, Merced, CA 95343, United States of America; 5 Johns Hopkins School of Medicine, Baltimore, MD 21205, United States of America; 6 Columbia University, NYS Psychiatric Institute, New York, NY 10032, United States of America; University of Padova, ITALY

## Abstract

**Background:**

Individuals with a history of suicide attempt have a high risk for subsequent labour market marginalization. This study aimed at assessing the effect of individual and parental factors on different measures of marginalization.

**Methods:**

Prospective cohort study based on register linkage of 5 649 individuals who in 1994 were 16–30 years old, lived in Sweden and were treated in inpatient care for suicide attempt during 1992–1994. Hazard ratios (HRs) for labour market marginalization defined as long-term unemployment (>180 days), sickness absence (>90 days), or disability pension in 1995–2010 were calculated with Cox regression.

**Results:**

Medical risk factors, particularly any earlier diagnosed specific mental disorders (e.g., schizophrenia: HR 5.4 (95% CI: 4.2, 7.0), personality disorders: HR 3.9, 95% CI: 3.1, 4.9), repetitive suicide attempts (HR 1.6, 95% CI: 1.4, 1.9) were associated with a higher relative risk of disability pension. Individual medical factors were of smaller importance for long-term sickness absence, and of only marginal relevance to long-term unemployment. Country of birth outside Europe had an opposite effect on disability pension (HR 0.6, 95% CI: 0.4, 0.8) and long-term unemployment (HR 1.5, 95% CI: 1.3, 1.8). Female sex was positively correlated with long-term sickness absence (HR 1.6, 95% CI: 1.4, 1.7), and negatively associated with long-term unemployment (HR: 0.8, 95% CI: 0.7, 0.9).

**Conclusions:**

As compared to disability pension, long-term sickness absence and unemployment was more strongly related to socio-economic variables. Marginalization pathways seemed to vary with migration status and sex. These findings may contribute to the development of intervention strategies which take the individual risk for marginalization into account.

## Introduction

Disability pension and sickness absence have increased in Sweden and other member countries of the Organisation for Economic Co-operation and Development (OECD) over the last decades, particularly in young people [1,2]. Mental disorders are now the predominant causes of leaving the labour market or never entering it [1,3]. Also, unemployment has increased among young people in Sweden and throughout Europe and North America, and individuals with mental disorders are likely to have even more difficulties establishing themselves on the labour market as compared to individuals without functional disabilities [4–6].

Social insurance policies such as sickness absence and disability pensions are intended to provide financial support for individuals with impaired work capacity due to disease or injury [7]. Due to the individual consequences and the tremendous costs involved for the society, the avoidance of unnecessary labour market marginalization as indicated by disability pension, long-term sickness absence, or long-term unemployment, and the reintegration of individuals into the labour market in accordance with functional capacity have been established as main goals of labour market policies. Absence from the labour market means a considerable loss of productivity [1,2], and may be linked to negative health effects in affected individuals, including a lack of meaning [8], reduced social integration, physical inactivity, sleep disturbances and low self-ratings of psychological well-being [9]. As already noted by Durkheim and replicated in several studies, social integration is one of the most powerful protective factors for suicide [10].

Suicide attempt can be viewed as one of the most detrimental consequences of mental disorders [11], and constitutes a considerable public health problem in many European countries, among them Sweden [4,12]. In recent studies, it was shown that suicide attempt may increase the risk of subsequent social welfare receipt independent of the effect of mental disorders [13,14].

Despite the magnitude of this problem and the consensus on the need to address it, studies on determinants of labour market marginalization in young people with suicide attempts are sparse. One study found that young suicide attempters, compared to non-attempters had significantly longer durations of unemployment later on in life, as well as a longer duration of welfare benefits [14]. With regard to different measures of labour market marginalization, suicide attempters had a higher risk of long-term sickness absence, long-term unemployment and particularly disability pension [13]. This study also revealed a dose-response relationship between the number of suicide attempts and the risk of disability pension, suggesting that individuals with higher psychiatric severity as indicated by repetitive suicide attempt hospitalizations were more likely to be granted disability pension. No such relationship was present for long-term unemployment.

It is currently unknown if the determinants of labour market marginalization vary across various labour market outcomes such as disability pension, long-term sickness absence and long-term unemployment. An earlier study showed that a diagnosis of schizophrenia and past violent suicide attempts were risk factors for subsequent sickness absence and disability pension in young suicide attempters [15], but this study had a small sample size and was conducted more than 40 years ago. Knowledge about differences between different measures of labour market marginalization is essential to address labour market marginalization in its various forms. Due to the dependence of disability pensions and sickness absences on medical assessments of work capacity, medically registered characteristics (e.g. repetitive suicide attempts, specific psychiatric diagnosis) are likely stronger determinants for these outcomes than for unemployment. On the other hand, the risk for unemployment might be greater in cases of socio-economic disadvantage. These patterns may further differ with regard to sex [2,7,16,17].

This study aimed to 1) investigate determinants of labour market marginalization (i.e., disability pension, long-term sickness absence, or long-term unemployment) in young suicide attempters, focusing on aspects related to the suicide attempt, medical risk factors, and social characteristics and 2) identify potential sex differences in the association between the specific risk factors and the three outcome measures.

## Material and Methods

The baseline study population comprised all individuals alive, resident in Sweden, between 16 and 30 years of age on December 31^st^, 1994, who did not have disability pension at baseline, with at least one parent born in Sweden and treated in inpatient care following attempted suicide during the three years preceding study entry, i.e. 1992, 1993 or 1994 (N = 5 649).

Suicide attempts were coded in accordance with the International Classification of Diseases 10th Revision (ICD-10). Among the 5 649 individuals with inpatient care due to attempted suicide between 1992 and 1994 when aged 14 to 30 years, 4 434 (78.5%) were determined suicide attempts, and 1 215 (21.5%) were of undetermined intent. In accordance with earlier studies both events of determined intent (X60-84) and of undetermined intent (Y10-34) according to the ICD-10 were combined. This procedure intends to limit temporal and geographic differences in ascertainment [13,18,19]. A sensitivity analysis of events of determined and undetermined intent showed that both codes were comparable. Hereafter the combined measure is referred to as “suicide attempt”.

Register data was available for each individual retrospectively and prospectively up to 31^st^ December 2010. Statistics Sweden provided data on age, sex, country of birth, education, and year and length of unemployment. Data on dates of sickness absence and disability pension from 1994 and onwards were derived from the Social Insurance Agency. Information on date and diagnosis of inpatient care starting from 1973 and the date and cause of death from 1961 and onwards were obtained from the National Board of Health and Welfare. The multi-generation register was used to identify the parents of the index subjects.

### Individual and parental determinants

Characteristics of index subjects include the repetition of suicide attempt hospitalizations, mental (diagnosis-specific) and somatic inpatient care, and socio-demographics (i.e., country of birth, area or residence, sex, age, educational level).

The following classification was used with regard to diagnosis-specific mental disorders: Schizophrenia and non-affective psychoses (F20-F29); affective disorders (F30-39); substance abuse disorders (F10-F19); neurotic, stress-related and somatoform disorders (F40-F48); organic disorders including mental retardation (F00-F09, F70-79); behavioural, emotional and developmental disorders (F50-59, F61-F69, F80-F89, F90-F99); personality disorders (F60) (Table 1).

**Table 1:** Descriptive statistics of individuals with inpatient care due to suicide attempt (1994–96).

Characteristics	All		Disability pension		Sickness absence (> 90 days)		Unemployment (>180 days)	
	n	%	n	%	n	%	n	%
***Index persons' sociodemographic characteristics***								
*Sex*								
Women	3567	63.1	959	65.6	1908	69.6	1519	58.6
Men	2082	36.9	502	34.4	832	30.4	1071	41.4
*Area of residence*^*1*^								
Small cities/villages	1554	27.5	415	28.4	783	28.6	738	28.5
Medium-sized cities	1992	35.3	519	35.5	963	35.1	961	37.1
Big cities	2103	37.2	525	35.9	994	36.3	891	34.4
*Age group*, *years*								
21–30 years	3665	64.9	1149	78.6	1975	72.1	1798	69.4
16–20	1984	35.1	312	21.4	765	27.9	792	30.6
*Country of birth*								
Rest of the world	318	5.6	45	3.1	143	5.2	184	7.1
Other EU25	65	1.2	17	1.2	21	0.8	31	1.2
Other Northern Europe	170	3.0	56	3.8	88	3.2	79	3.1
Sweden	5096	90.2	1343	91.9	2488	90.8	2296	88.6
*Educational level (years)*								
Low (≤9 years)	2565	45.4	372	25.5	1220	44.5	1201	46.4
Medium (10–12 years)	2538	44.9	708	48.5	1312	47.9	1199	46.3
High (≥12 years)	287	5.1	277	19.0	132	4.8	85	3.3
Missing information	259	4.6	104	7.1	76	2.8	105	4.1
*Previous number of suicide attempts*								0.0
>3	607	10.7	289	19.8	339	12.4	260	10.0
2–3	990	17.5	300	20.5	547	20.0	431	16.6
1	4052	71.7	872	59.7	1854	67.7	1899	73.3
*Main diagnosis at earlier psychiatric inpatient hospital care*^*2*^								0.0
Schizophrenia	103	1.8	68	4.7	49	1.8	43	1.7
Affective disorders	270	4.8	109	7.5	144	5.3	105	4.1
Substance abuse	607	10.7	218	14.9	290	10.6	306	11.8
Neurotic, somatoform and stress-related disorders	848	15.0	297	20.3	460	16.8	388	15.0
Organic or retardation	19	0.4	8	0.5	8	0.3	11	0.4
Developmental disorders	422	7.5	158	10.8	222	8.1	167	6.4
Personality disorders	163	2.9	90	6.2	79	2.9	58	2.2
No previous inpatient care	3217	56.9	513	35.1	1488	54.3	1512	58.4
*Earlier somatic inpatient care*								
Yes	4077	72.2	1176	80.5	2510	91.6	1901	73.4
No	1572	27.8	285	19.5	230	8.4	689	26.6
***Parental characteristics***								
*Educational level (years)*^*3*^								
Low (≤9 years)	1364	24.1	372	25.5	709	25.9	654	25.3
Medium (10–12 years)	2691	47.6	708	48.5	1333	48.6	1271	49.1
High (≥12 years)	1211	21.4	277	19.0	516	18.8	467	18.0
Missing information	383	6.8	104	7.1	182	6.6	198	7.6
*Psychiatric inpatient care*								
At least one parent	1365	24.2	440	30.1	703	25.7	706	27.3
None	4274	75.8	1021	69.9	2037	74.3	1884	72.7
*Disability pension*								
At least one parent	1319	23.3	456	31.2	705	25.7	645	24.9
None	4330	76.7	1005	68.8	2035	74.3	1945	75.1
*Somatic inpatient care*								
At least one parent	5150	91.2	1347	92.2	2510	91.6	2350	90.7
None	499	8.8	114	7.8	230	8.4	240	9.3
*Suicide attempt*								
At least one parent	649	11.5	187	12.8	318	11.6	314	12.1
None	5000	88.5	1274	87.2	2422	88.4	2276	87.9
*Death*								
Suicide—at least one parent	119	2.1	34	2.3	50	1.8	54	2.1
Other death—at least one parent	472	8.4	157	10.7	245	8.9	236	9.1
None	5058	89.5	1270	86.9	2445	89.2	2300	88.8

^1^ Area of residence: big cities: Stockholm, Göteborg, and Malmö; medium-sized cities: cities with more than 90 000 inhabitants within 30 km distance from the centre of the city; small cities/villages [20].

^2^ See methods section for ICD-10 codes included.

^3^ Paternal or maternal education, whichever highest.

Parental characteristics included parental disability pension, mental and somatic inpatient care, suicide attempt, suicide, death other than suicide, as well as parental educational level (Table 1). Paternal or maternal education, whichever was highest was used, in accordance with previous studies [13]. For suicide, events of determined and undetermined intent were combined in the same way as for suicide attempts (see above).

All individual variables and parental markers were measured from the start of the respective register (see previous paragraph) up until the year before the index event for individuals with suicide attempt.

The socio-demographic characteristics (age, sex) of index persons and parents were measured on December 31st 1994.

### Outcome measures

Three different outcome measures were analysed: 1) disability pension, 2) long-term sickness absence (more than 90 registered days with sickness absence during one calendar year), and 3) long-term unemployment (more than 180 registered days with unemployment benefits during one calendar year).

All residents with a permanently reduced work capacity due to injury or disease can be granted permanent disability pension. Since 2003, temporary disability pension (“aktivitetsersättning”) can be granted for young individuals between 19 and 29 years of age in case of reduced work capacity or in case that compulsory education is not completed at 19 years [7]. Sick pay is paid by the respective employer for the first 2 weeks, and there is one qualifying day (more for self-employed) without benefits. It is generally granted temporarily. After seven days of self-certification, a physician certificate is required. Sickness absence amounts to a capped 80% of lost income, disability pension to approximately 65%.

All individuals who live in Sweden, aged over 16 years and have an income from work or unemployment benefits are eligible for sickness benefits by the Social Insurance Agency if they have a reduced work capacity due to disease or injuries.

In order to be eligible for unemployment benefits, the individual needs to be unemployed, able to work, and ready to accept work that is offered. In addition, prior to becoming unemployed the applicant shall have worked at least six months [21]. The data used here cover benefits paid by the Social Insurance Agency [7].

### Statistical methods

For all individual and parental determinants, missing values were coded as a separate category and included in the analysis. Cox regression with time dependent covariates was used in order to adjust for changes in individual risk factors during follow-up. Time dependent covariates measured during follow-up included suicide attempt, inpatient care due to mental/somatic diagnoses and inclusion in the labour market (as indicated by a minimum annual salary). The following additional time-dependent variables during follow-up were considered for specific outcomes as reported in brackets: long-term unemployment/year (disability pension, long-term sickness absence) and long-term sickness absence (unemployment). All time-dependent covariates were dichotomized and measured annually.

Follow up lasted for as long as 16 years (1995–2010). Censoring was due to emigration to a foreign country, death, or end of follow up, whichever occurred first. In the analyses of unemployment and sickness absence as outcome measures, censoring was also due to disability pension because individuals with disability pension are not eligible for unemployment or sickness absence benefits.

Interaction of sex with the other risk factors was tested with the partial likelihood ratio test. If there was a significant interaction effect (p<0.05), analyses were stratified by sex.

### Ethical statement

Ethical approval for this study was obtained from the Regional Ethical Review Board, Karolinska Institutet, Stockholm (review number 2007/762-31). The ethical review board approved the study and waived the requirement that informed consent of research subjects should be obtained. The authors assert that all procedures contributing to this work comply with the ethical standards of the relevant national and institutional committees on human experimentation and with the Helsinki Declaration of 1975, as revised in 2008.

## Results

Of individuals with a history of suicide attempts (n = 5 649), 4052 (71.0%) had one attempt, 990 (17.5%) had 2–3 registered attempts, and 607 (10.7%) had >3 attempts. A clear majority of attempts were due to poisoning (n = 4 897; 86.7%). Cutting (n = 325; 5.8%) was the second most frequent method used. During the follow-up 194 (3.4%) individuals died by suicide and 150 (2.7%) by other causes of death.

Table 1 shows descriptive characteristics of the total study population and for each outcome variable. Suicide attempters were often female (63.1%), in the age group over 20 years (64.9%), and had low (45.4%) or medium (44.9%) educational level. Among suicide attempters with previous psychiatric inpatient care (43.1%), neurotic, somatoform and stress-related disorders (34.8%) were the most frequent diagnostic groups, followed by substance abuse (24.8%) and developmental disorders (17.4%). At least one parent had received psychiatric inpatient care or disability pension in 24.2% and 23.3% of suicide attempters, respectively. Parental education was low or medium in 24.1% and 47.6% of suicide attempters, respectively. In total, 2.1% of attempters had lost a parent due to suicide, and 8.4% to other causes than suicide.

### Disability pension

In the crude analyses, granting of disability pension was positively associated with older age (>20 years, HR 2.3, 95% CI: 2.0, 2.6), negatively associated with country of birth outside the European Union (HR 0.5, 95% CI: 0.4, 0.7), and the hazard ratio increased with number of suicide attempts reaching a three-fold HR for individuals with three or more attempts. Suicide attempters with previous psychiatric inpatient care had increased risk for disability pension, with HRs being highest for schizophrenia (HR 7.2, 95% CI: 5.6, 9.3), followed by personality disorders (HR 5.2, 95% CI: 4.1, 6.5). Earlier somatic inpatient care was associated with an HR of 1.7 (95% CI: 1.5, 1.9). Among the parental characteristics, low educational level (HR 1.2, 95% CI: 1.0, 1.4), psychiatric inpatient care (HR 1.4, 95% CI: 1.3, 1.6), disability pension (HR 1.6, 95% CI: 1.5, 1.8), and parental non-suicidal death (HR 1.4, 95% CI: 1.2, 1.6) were associated with a higher risk for disability pension in offspring (Table 2).

**Table 2:** Crude hazard ratios (HR) and 95% confidence intervals (CI) for the risk of unemployment (>180 days), sickness absence (>90 days), and disability pension in 1995–2010 in young suicide attempters in 1992–94.

	Disability pension		Sickness absence (> 90 days)		Unemployment (>180 days)	
	HR	95% CI	HR	95% CI	HR	95% CI
***Index persons' sociodemographic characteristics***						
*Sex*						
Women	1.10	0.98, 1.22	1.43	1.31, 1.55	0.75	0.69, 0.81
Men	1		1		1	
*Area of residence*^*1*^						
Small cities/villages	1.04	0.91, 1.18	1.05	0.96, 1.14	1.15	1.02, 1.27
Medium-sized cities	1.05	0.93, 1.18	1.07	0.98, 1.18	1.20	1.09, 1.31
Big cities	1		1		1	
*Age group*, *years*						
21–30 years	2.27	2.00, 2.57	1.76	1.62, 1.91	1.39	1.28, 1.51
16–20 years	1		1		1	
*Country of birth*						
Rest of the world	0.51	0.38, 0.68	0.88	0.74, 1.04	1.39	1.20, 1.61
Other EU25	1.07	0.66, 1.72	0.59	0.38, 0.90	1.20	0.84, 1.71
Other Northern Europe	1.40	1.07, 1.83	1.18	0.95, 1.46	1.06	0.85, 1.33
Sweden	1		1		1	
*Educational level (years)*						
Low (≤9 years)	1.17	0.91, 1.50	1.02	0.85, 1.22	1.76	1.41, 2.19
Medium (10–12 years)	1.02	0.79, 1.31	1.16	0.97, 1.39	1.81	1.46, 2.26
High (≥12 years)	1		1		1	
Missing information	1.34	0.97, 1.86	0.54	0.40, 0.71	1.41	1.06, 1.88
*Previous number of suicide attempts*						
>3	2.92	2.56, 3.34	1.50	1.33,1.68	0.91	0.80, 1.04
2–3	1.55	1.36, 1.76	1.36	1.24, 1.50	0.93	0.84, 1.04
1	1		1		1	
*Main diagnosis at earlier psychiatric inpatient hospital care*^*2*^						
Schizophrenia	7.19	5.58, 9.26	1.24	0.93, 1.65	0.88	0.65, 1.18
Affective disorders	3.38	2.74, 4.15	1.39	1.17, 1.65	0.81	0.67, 0.99
Substance abuse	2.85	2.43, 3.34	1.22	1.08, 1.38	1.17	1.03, 1.32
Neurotic, somatoform and stress-related disorders	2.63	2.28, 3.03	1.36	1.22, 1.51	0.99	0.88, 1.10
Organic or retardation	4.05	2.02, 8.15	1.04	0.52, 2.08	1.28	0.71, 2.31
Developmental disorders	2.83	2.37, 3.38	1.24	1.07, 1.42	0.80	0.69, 0.94
Personality disorders	5.19	4.14, 6.49	1.18	0.94, 1.49	0.73	0.56, 0.95
No previous inpatient care	1		1		1	
*Earlier somatic inpatient care*						
Yes	1.69	1.49, 1.93	1.46	1.33, 1.59	1.10	1.01, 1.20
None	1		1		1	
***Parental characteristics***						
*Educational level (years)*^*3*^						
Low (≤9 years)	1.21	1.03, 1.41	1.35	1.21, 1.51	1.34	1.19, 1.51
Medium (10–12 years)	1.15	1.00, 1.32	1.23	1.11, 1.36	1.30	1.17, 1.44
High (≥12 years)	1		1		1	
Missing information	1.22	0.98, 1.53	1.22	1.03, 1.44	1.53	1.30, 1.81
*Psychiatric inpatient care*						
At least one parent	1.41	1.27, 1.58	1.14	1.05, 1.24	1.25	1.14, 1.36
None	1		1		1	
*Disability pension*						
At least one parent	1.64	1.47, 1.83	1.28	1.18, 1.40	1.15	1.05, 1.25
None	1		1		1	
*Somatic inpatient care*						
At least one parent	1.16	0.96, 1.40	1.10	0.96, 1.26	0.95	0.83, 1.08
None	1		1		1	
*Suicide attempt*						
At least one parent	1.16	1.00, 1.35	1.04	0.92, 1.17	1.10	0.98, 1.24
None	1		1		1	
*Death*						
Suicide—at least one parent	1.25	0.89, 1.75	0.91	0.69, 1.20	1.03	0.79, 1.35
Other death—at least one parent	1.39	1.18, 1.64	1.17	1.02, 1.33	1.19	1.04, 1.36
None	1		1		1	1

^1^ Area of residence: big cities: Stockholm, Göteborg, and Malmö; medium-sized cities: cities with more than 90 000 inhabitants within 30 km distance from the centre of the city; small cities/villages [20].

^2^ See methods section for ICD-10 codes included

^3^ Paternal or maternal education, whichever highest.

In the fully adjusted model, female sex (HR 1.2, 95% CI: 1.1, 1.3), older age (HR 1.9, 95% CI: 1.9, 2.5) and low educational level (HR 1.5, 95% CI: 1.2, 1.9) remained positively associated with subsequent disability pension, while country of birth outside the European Union decreased the relative risk of disability pension (HR 0.6, 95% CI: 0.4, 0.8).

Repeated suicide attempts were associated with a higher risk of disability pension (3 or more attempts: HR 1.6, 95% CI: 1.4, 1.9). Schizophrenia (HR 5.4, 95% CI: 4.2, 7.0) and personality disorders (HR 3.9, 95% CI: 3.1, 4.9) remained the diagnoses with the largest risk estimates related to disability pension. Earlier somatic inpatient care (HR 1.2, 95% CI: 1.1, 1.4) and parental disability pension (HR 1.4, 95% CI: 1.2, 1.5) were also significantly associated with disability pension (Table 3).

**Table 3:** Adjusted hazard ratios (HR) and 95% confidence intervals (CI) for the risk of unemployment (>180 days), sickness absence (>90 days), and disability pension in 1995–2010 in relation young suicide attempters in 1992–94.

	Disability pension		Sickness absence (> 90 days)		Unemployment (>180 days)	
	HR	95% CI	HR	95% CI	HR	95% CI
***Index persons' sociodemographic characteristics***						
*Sex*						
Women	1.20	1.07, 1.34	1.56	1.43, 1.70	0.80	0.74, 0.87
Men	1		1		1	
*Area of residence*^*1*^						
Small cities/villages	1.02	0.90, 1.17	1.11	1.01, 1.23	1.17	1.07, 1.28
Medium-sized cities	1.01	0.89, 1.14	1.07	0.97, 1.17	1.12	1.02, 1.24
Big cities	1		1		1	
*Age group*, *years*						
21–30 years	2.14	1.86, 2.47	1.70	1.54, 1.87	1.52	1.38, 1.67
16–20 years	1		1		1	
*Country of birth*						
Rest of the world	0.61	0.44, 0.83	1.09	0.88, 1.35	1.50	1.27, 1.77
Other EU25	1.21	0.74, 1.97	0.70	0.45, 1.08	1.25	0.87, 1.78
Other Northern Europe	1.19	0.91, 1.56	1.02	0.85, 1.23	0.96	0.76, 1.20
Sweden	1		1		1	
*Educational level (years)*						
Low (≤9 years)	1.49	1.15, 1.94	1.11	0.92, 1.34	1.79	1.42, 2.24
Medium (10–12 years)	1.13	0.87, 1.46	1.16	0.96, 1.39	1.75	1.40, 2.19
High (≥12 years)	1		1		1	
Missing information	2.18	1.54, 3.08	0.69	0.51, 0.92	1.48	1.10, 2.00
*Previous number of suicide attempts*						
3+	1.61	1.39, 1.86	1.22	1.10, 1.34	0.91	0.79, 1.05
2–3	1.09	0.95, 1.25	1.19	1.05, 1.35	0.95	0.85, 1.06
1	1		1		1	
*Main diagnosis at earlier psychiatric inpatient hospital care*^*2*^						
Schizophrenia	5.41	4.18, 7.00	1.02	0.77, 1.36	0.78	0.57, 1.06
Affective disorders	2.68	2.16, 3.31	1.16	0.97, 1.40	0.80	0.66, 0.98
Substance abuse	2.18	1.85, 2.57	1.07	0.94, 1.23	1.00	0.88, 1.14
Neurotic, somatoform and stress-related disorders	2.00	1.72, 2.32	1.11	0.99, 1.24	0.93	0.83, 1.05
Organic or retardation	3.38	1.67, 6.82	1.00	0.50, 2.01	1.22	0.67, 2.22
Developmental disorders	2.37	1.96, 2.86	1.15	0.99, 1.33	0.87	0.74, 1.03
Personality disorders	3.85	3.06, 4.86	0.92	0.73, 1.16	0.67	0.52, 0.88
No previous inpatient care	1		1		1	
*Earlier somatic inpatient care*						
Yes	1.22	1.06, 1.39	1.27	1.16, 1.39	1.06	0.97, 1.16
None	1		1		1	
***Parental characteristics***						
*Educational level (years)*^*3*^						
Low (≤9 years)	1.05	0.91, 1.21	1.22	1.09. 1.38	1.18	1.06, 1.32
Medium (10–12 years)	0.93	0.79, 1.10	1.16	1.05, 1.29	1.14	1.01, 1.30
High (≥12 years)	1		1		1	
Missing information	0.93	0.72, 1.21	1.14	0.94, 1.38	1.26	1.04, 1.52
*Psychiatric inpatient care*						
At least one parent	1.11	0.98, 1.26	1.06	0.96, 1.17	1.23	1.12, 1.36
None	1		1		1	
*Disability pension*						
At least one parent	1.35	1.20, 1.52	1.12	1.02, 1.23	0.99	0.90, 1.09
None	1		1		1	
*Somatic inpatient care*						
At least one parent	0.99	0.81, 1.20	1.02	0.89, 1.18	0.97	0.84, 1.11
None	1		1		1	
*Suicide attempt*						
At least one parent	0.88	0.74, 1.04	0.92	0.81, 1.05	0.96	0.84, 1.09
None	1		1		1	
*Death*						
Suicide—at least one parent	1.11	0.79, 1.57	0.86	0.65, 1.14	0.92	0.70, 1.21
Other death—at least one parent	1.16	0.96, 1.40	1.03	0.89, 1.20	1.03	0.89, 1.20
None	1		1		1	

^1^ Area of residence: big cities: Stockholm, Göteborg, and Malmö; medium-sized cities: cities with more than 90 000 inhabitants within 30 km distance from the centre of the city; small cities/villages [20].

^2^ See methods section for ICD-10 codes included.

^3^ Paternal or maternal education, whichever highest.

Note: Adjustment was for all other variables in the table. In addition, the following time-dependent covariates were adjusted for: suicide attempt, inpatient care due to mental/somatic diagnoses and inclusion in the labour market (minimum annual salary). In addition: (specific outcomes are mentioned in brackets): long-term unemployment/year (for outcome disability pension, sickness absence) and long-term sickness absence (for outcome unemployment). All time-dependent covariates were dichotomized and controlled for each year of follow-up.

### Sickness absence ≥90 days

In the crude analyses, long-term sickness absence was positively associated with female sex (HR 1.4, 95% CI: 1.3, 1.6) and older age (>20 years, HR 1.8, 95% CI: 1.6, 1.9), and negatively associated with country of birth in the European Union but outside of Scandinavia (HR 0.6, 95% CI: 0.4, 0.9). The hazard ratio increased in a dose-response pattern with number of suicide attempts, reaching a 1.5-fold HR for individuals with three or more attempts (HR 95% CI: 1.3, 1.7). Most psychiatric diagnostic groups of individuals with previous psychiatric inpatient care had increased HR for long-term sickness absence, with HRs being highest for affective disorders (HR 1.4, 95% CI: 1.2, 1.7), followed by developmental disorders (HR 1.2, 95% CI: 1.1, 1.4). Earlier somatic inpatient care was associated with a HR of 1.5 (95% CI: 1.3, 1.6). Among the parental characteristics, low educational level (HR 1.4, 95% CI: 1.2, 1.5), psychiatric inpatient care (HR 1.1, 95% CI: 1.1, 1.2), disability pension (HR 1.3, 95% CI: 1.2, 1.4), and parental non-suicidal death (HR 1.2, 95% CI: 1.0, 1.3) were all associated with a higher risk for sickness absence in offspring (Table 2).

In the fully adjusted model, female sex (HR 1.6, 95% CI: 1.4, 1.7), older age (HR 1.7, 95% CI: 1.5, 1.9) and residence in small cities or villages (HR 1.1, 95% CI: 1.0, 1.2) were positively associated with subsequent sickness absence. Repeated suicide attempts were associated with a higher risk (three or more attempts: HR 1.2, 95% CI: 1.1, 1.3), and there was no additional risk from previous psychiatric diagnoses on the risk of long-term sickness absence. Earlier somatic inpatient care (HR 1.3, 95% CI: 1.2, 1.4) and parental disability pension (HR 1.1, 95% CI: 1.0, 1.2) had some effect on long-term sickness absence in offspring, and sickness absence was also associated with parental education, with a 1.2-fold risk (95% CI: 1.1, 1.4) for offspring of parents with low educational level (Table 3).

An interaction with sex and repetitive suicide attempts was found with regard to subsequent sickness absence. Men with two and more than two suicide attempts had higher HRs (1.4 (1.2–1.7); 1. 5 (1.1–1.9) for subsequent sickness absence, respectively. Related estimates for women were 1.1 (1.0–1.3) and 1.1 (1.0–1.3).

There was an additional interaction with sex and previous mental diagnosis-specific inpatient care with regard to subsequent sickness absence. Men had generally higher HRs than women. For example, the HRs and CIs for inpatient care due to substance abuse and behavioural disorders for males were 1.2 (1.0–1.5) and 1.4 (1.0–2.1), respectively. The corresponding estimates among females were 0.9 (0.8–1.1) and 1.1 (0.9–1.3), respectively.

### Unemployment ≥180 days

In the crude analyses, long-term unemployment was negatively associated with female sex (HR 0.8, 95% CI: 0.7, 0.8), and positively associated with older age (>20 years, HR 1.4, 95% CI: 1.3, 1.5), residence in villages (HR 1.2, 95% CI: 1.0, 1.3) or medium-sized cities (HR 1.2, 95% CI: 1.1, 1.3), country of birth outside of the European Union (HR 1.4, 95% CI: 1.2, 1.6), and low educational level (HR 1.8 95% CI: 1.4, 2.2). Among previous psychiatric medical diagnostic groups, only a diagnosis of substance abuse (HR 1.2, 95% CI: 1.0, 1.3) was significantly associated with long-term unemployment. Earlier somatic inpatient care (HR 1.1, 95% CI: 1.0, 1.2), and parental characteristics including low educational level (HR 1.3, 95% CI: 1.2, 1.5), psychiatric inpatient care (HR 1.3, 95% CI: 1.1, 1.4), disability pension (HR 1.2, 95% CI: 1.1, 1.3) and non-suicidal death (HR 1.2, 95% CI: 1.0, 1.4) were also associated with long-term unemployment in the crude analyses (Table 2).

In the adjusted model, female sex was negatively associated with subsequent long-term unemployment (HR 0.8, 95% CI: 0.7, 0.9). Residence in villages (HR 1.2, 95% CI: 1.1, 1.3) or medium-sized cities (HR 1.1, 95% CI: 1.0, 1.2), country of birth outside the European Union (HR 1.5, 95% CI: 1.3, 1.8) and low (HR 1.8, 95% CI: 1.4, 2.2) or medium educational level (HR 1.7, 95% CI: 1.4, 2.2) remained significant predictors of subsequent long-term unemployment. Also low (HR 1.2, 95% CI: 1.1, 1.3) or medium (HR 1.1, 95% CI: 1.0, 1.3) parental educational level, and parental psychiatric inpatient care (HR 1.2, 95% CI: 1.1, 1.4) remained associated with later long-term unemployment (Table 3).

## Discussion

### Main findings

We examined the association between various medical, socio-demographic, and parental risk factors among persons who had inpatient care due to suicide attempt with subsequent labour market marginalization, which was defined as long-term unemployment, long-term sickness absence or granting of disability pension. Medical risk factors were most important for the subsequent granting of disability pension and also for long-term sickness absence. With regard to long-term unemployment, medical risk factors were of marginal importance. In contrast, all socio-demographic factors were associated with higher risk for unemployment. This included male sex and non-European migrant status. On the other hand, male sex and birth outside Europe were associated with decreased risk of being granted disability pension. Interaction tests revealed that men with previously diagnosed mental disorders or repeated attempts had higher risk of long-term sickness absence than women.

This study revealed clear variation in the relevance of socio-demographic and medical risk factors for various markers of labour market marginalization. We found that repeated suicide attempts and somatic comorbidity in young suicide attempters were predictive for subsequent sickness absence and disability pension but not for unemployment. Previous inpatient care due to mental disorders was only predictive of subsequent disability pension. Consistent with earlier studies, schizophrenia and personality disorders were most strongly associated with disability pension [15,22]. Potential explanations include that the medical severity of the underlying mental disorder, repetitive attempts and somatic comorbidity are likely to have a stronger effect on outcomes which are based upon a medical assessment (sickness absence, disability pension) as compared to other outcomes independent from assessments such as unemployment. Still, another potential explanation for the lower relevance of medical factors in long-term unemployment may be due to a selection of healthier individuals into the group at risk for unemployment. The healthy worker effect suggests that individuals who are sicker are less likely to enter the labour market [23], and more likely to leave it prematurely e.g. due to disability pension or death. The sicker individuals may therefore be less likely to be at risk for unemployment and a competing risk scenario may be present.

Being born in a country outside Europe was associated with decreased risk of disability pension but increased risk for unemployment. This finding may signal that immigrants with suicide attempts who were born outside Europe enter a labour market marginalization track that typically involves long-term unemployment, but not granting of disability pension. In contrast to this finding, previous studies reported an increased risk of disability pension for immigrants from outside the EU who experienced sickness absence due to stress-related mental disorders or depressive episodes [24,25]. Beside the fact that our study population was defined differently as individuals with suicide attempt, the study population in our study was also younger and follow-up was considerably longer. Potential social and cultural differences in the access to healthcare, treatment, evaluation of a disability pension or in the process of a granting disability pensions for suicide attempters with different ethnic and cultural background might be alternative explanations for this finding [26,27]. Future studies are warranted to elucidate mechanisms behind the lower risk of disability pension and the higher risk of unemployment among non-European migrants with suicide attempt.

Lower socio-economic status and older age was predictive for all outcome measures, though to varying degrees. This is in line with previous findings, that labour market marginalization is socially determined [28,29]. Living in rural areas was predictive of sickness absence and unemployment. Similar findings were reported in sickness absentees due to stress-related mental disorder and outpatients due to depressive episode with regard to higher relative risk of subsequent disability pension [24,25]. Regional differences in access to the labour market and health care as well as differences in rehabilitation options in rural areas might explain this finding.

Women had higher risk of being granted disability pension or long-term sickness absence, whereas men had a higher relative risk for long-term unemployment which is in line with previous studies in individuals with sickness absence or outpatient care due to mental disorders [25]. Men had a higher risk for sickness absences in case of repetitive suicide attempts and previous inpatient care due to mental disorders than women. Potential explanations for these gender differences include the lower help-seeking behaviour among men [30], resulting in under diagnosis and under treatment of mental disorders. Both inpatient care due to mental disorders and repetitive suicide attempts were considerably more prevalent among women compared to men. Men with inpatient care due to mental disorders or repetitive suicide attempts may therefore be characterised by a higher medical severity as compared to women.

In contrast, parental disability pension was associated with a higher risk of disability pension and long-term sickness absence in offspring suicide attempters. Both intra-familial morbidity and social patterns that may influence granting of disability pension may play a role in this finding. A recent study identified low labour market participation in the parents as risk factors for sickness absence in the offspring [31]. Parental inpatient care due to mental disorders was the only parental factor associated with an increased risk of long-term unemployment. Parental psychiatric morbidity could be viewed as a medical risk factor or a marker of psychosocial adversity during childhood, including but not limited to less beneficial child rearing styles and less stable parent-child relationships [19].

### Strengths and limitations

Strengths of the present study include its prospective design and the use of national register data which have practically no dropout rate. Evaluations of several registers have shown high data quality. For example, 1.2% and 0.8% missing main diagnoses were reported for the National Patient Register and the Causes of Death Register, respectively [32]. Data on sickness absence and disability pension were obtained from economically based registers which indicate compensation received. This type of register is generally considered to be of good quality [7]. Recall bias which is a strong source of bias in studies from clinical samples, was not relevant to the present evaluation.

With regard to study limitations, the present study was based on individuals who were hospitalized for suicide attempts. As it is known that only a quarter of suicide attempters receive inpatient care, the present findings can only be generalized to individuals who have more serious medical conditions that result in hospitalization [33]. Follow-up studies with non-hospitalized suicide attempters are warranted. Further, some individuals might not have received unemployment benefits despite being unemployed. However, in Sweden young unemployed adults who have never worked can have unemployment benefits and are thus included here. Another limitation is that the occurrence of unemployment, sickness absence, and disability pension is affected by regional and temporal changes in social insurance policies and in fluctuations in economic development [34,35]. Risk factors for the specific markers of labour market marginalization may also show some regional and temporal variations. Studies based on different birth cohorts and from different countries are needed in order to analyse potential differences in patterns.

## Conclusions

Labour market marginalization is not a homogenous construct, as evidenced by the different associations of medical and socio-demographic factors with disability pension, sickness absence and long-term unemployment. While medical factors played a detrimental role in the receipt of disability pension and sickness absence, their role in long-term unemployment was only marginal. Moreover, marginalization processes seemed to differ by sex and migration status, with birth country having a different effect on disability pension and long-term unemployment, and repeated suicide attempts having a stronger effect for men than women with regard to long-term sickness-absence. Because labour market marginalization could result in a worsening of risk for suicide due to social isolation and social marginalization, it would be ideal to be able to establish programs designed to keep those with labour market marginalization engaged in the labour market. These findings may contribute to the design of clinical and public health intervention strategies which have to take the identified individual variations in risk for marginalization into account.
